# Adenoviral vector mediated ferritin over-expression in mesenchymal stem cells detected by 7T MRI in vitro

**DOI:** 10.1371/journal.pone.0185260

**Published:** 2017-09-25

**Authors:** Hai-yang Dai, Rong He, Ying Zhang, Ren-hua Wu, Ye-yu Xiao

**Affiliations:** 1 Department of Medical Imaging, Huizhou Municipal Central Hospital, Huizhou, China; 2 Department of Medical Imaging, the 2^nd^ Affiliated Hospital of Shantou University Medical College, Shantou, China; Fraunhofer Research Institution of Marine Biotechnology, GERMANY

## Abstract

**Objective:**

The aim of the present work was to verify whether adenoviral vector mediated ferritin over-expression in mesenchymal stem cells could be detected by 7T MRI device, and to explore the relationship between ferritin content and MRI signal intensities.

**Methods:**

A recombined adenoviral vector (rAdV) encoding ferritin heavy chain (FTH1) subunit was specially designed for the aim of infecting bone marrow mesenchymal stem cells (BMSCs). Ferritin over-expression in BMSCs was determined by cell immunocytochemistry and the ferritin content in cells was determined by ELISA assay. BMSCs were subjected to cell viability, proliferation and multi-differentiation analyses as well as 7T MRI test using fast spin-echo pulse sequence. The R2 value andδR2 were calculated according to T2 mapping images.

**Results:**

As was confirmed by cell immunocytochemistry and ELISA assay, rAdV mediated ferritin was over-expressed in BMSCs. Ferritin over-expression did not interfere with stem cell viability or pluripotent differentiation but slowed cell proliferation. The R2 value of BMSCs_-FTH1_ vs control BMSCs from 1–4 weeks was16.65±1.28 s^-1^ vs 13.99±0.80 s^-1^, (t = 3.94, p = 0.004), 15.63±1.37 s^-1^ vs 13.87±0.83 s^-1^ (t = 2.47, p = 0.039), 15.53±0.88 s^-1^ vs 14.25±0.53 s^-1^ (t = 2.80, p = 0.023) and 14.61±1.28 s^-1^ vs 13.69±1.03 s^-1^ (t = 1.25, p = 0.24), respectively. δR2 gradually decreased from 1–4 weeks and the difference between the groups had statistical significance (F = 12.45, p<0.01).δR2 was positively correlated with OD value (r = 0.876, p<0.01) and ferritin concentration (r = 0.899, p<0.01) as determined by Pearson correlation.

**Conclusions:**

Our study confirms that ferritin could be over-expressed in BMSCs as a result of rAdV mediated infection and could be quantitatively detected by 7T MRI device. The differences in T2 signal intensities and R2 values stem from internal contrast generated by endogenous ferritin over-expression. The correlation between δR2, OD and ferritin concentration suggests that MRI can detect ferritin signal change accurately.

## Introduction

Bone marrow mesenchymal stem cells (BMSCs) are pluripotent progenitors and keep the ability of multi-differentiation [1–3]. Stem cell transplantation has shown great prospects in damage repair and tissue engineering, and can help avoid several ethical and technical issues [4]. At present, one problem hindering stem cell application in clinic is the lack of an appropriate way to monitor cell status after transplantation. For a long period of time, superparamagnetic iron oxides (SPIOs), which can decrease T2 relaxation time, has been considered as a novel exogenous contrast medium for stem cell labeling [5, 6]. In recent years, however, more and more studies doubted the validity of SPIOs in distinguishing surviving cells as it may be contaminated and confused by signals from other sources like hemoglobin, macrophages or dead cells [7, 8]. Besides, it was found that cell survival time may be overestimated by MRI when labeled with SPIOs [9]. In short, this well recognized exogenous contrast has shown some defects and limitations. Therefore, new strategies for cell labeling are badly in need.

Ferritin is a primary intracellular iron-storage protein, keeping irons in soluble and non-toxic form. It is a protein of 450 kDa containing 24 subunits and exists in all cell types. In vertebrate animals, the subunit consists of a light (L) and a heavy (H) chain of 19 kDa and 21 kDa, respectively[10]. The H-chain subunit, which has ferroxidase activity that promotes iron oxidation and incorporation, is the main regulator of ferritin activity and responsible for up-regulation of transferrin receptor and increases iron uptake. The L-chain lacks ferroxidase activity but promotes the nucleation and enables storage of iron atom inside the protein shell [11, 12]. Ferritin can shorten both T1 and T2 relaxation time, which lays the foundation for MRI contrast [13, 14]. However in wild type cells, the ferritin contents are too minimal to be detected even in ultra-high field MR devices. In order to be detected, ferritin gene should be over-expressed for the sake of triggering cellular response and augmenting iron uptake to the detectable threshold of MRI. It was reported that ferritin gene over-expression could transiently decrease intracellular iron concentration and lead to physiological compensation of augmenting iron uptake [14, 15]. Recent work by Liu confirmed that transgenic cells over-expressing ferritin gene are characterized by up-regulation of transferring receptors and increase in iron content [16]. Several studies have confirmed that endogenous ferritin over-expression could generate T2 contrast that is detectable on MRI device in breast cancer cells, glioma cells or neural cells [15, 17, 18]. These results indicate the potential use of ferritin as an endogenous reporter for detection by noninvasive MRI.

Employments of innovative labeling mediums in stem cells are badly in demand. The inherent merits of ferritin make it a good candidate for endogenous contrast. From this aspect, we designed this experiment. The aim of the present study is to verify the feasibility of ferritin serving as an endogenous contrast medium for BMSCs and figure out the relationship between ferritin content and MRI signal intensities in an ex-vivo condition. In the present work, we constructed a recombinant adenoviral vector (rAdV) encoding ferritin H-chain gene sequence (FTH1) to infect BMSCs. The construction of rAdV-FTH1 was confirmed by PCR and gene sequence analysis, while ferritin over-expression was confirmed by immunocytochemistry and ELISA assay. MR imaging of endogenous ferritin was determined by acquiring the T2 and R2 values of BMSCs suspended in argorse using a 7 Telsa MR device and T2-mapping sequence. Then we statistically analyzed the relationship between ferritin content and R2 values. Our results confirm that rAdV mediated ferritin gene could be over-expressed in BMSCs, and endogenous ferritin could be quantitatively detected by 7T MRI device. Our results also reveal that the changes of R2 values are positively correlated with ferritin contents. The MRI contrast was derived from endogenous ferritin over-expression, which may open possibilities for tracking or monitoring BMSCs by noninvasive MRI.

## Materials and methods

### Construction of rAdV-FTH1 plasmid

The AdV (pHBAd-MCMV-GFP) plasmid was purchased from Hanbio Company (Hanbio, Shanghai, China). It contains a Green Fluorescent Protein (GFP) gene and a multiple clone site. The FTH1 (GeneBank accession No.NP-034369.1) sequence, which contains 3000 bp of nucleotides, was synthesized by GenScript Company (GenScript USA Inc., Piscataway, USA), with 5’- and 3’- ends added. The construction work was accomplished with technical supports from Hanbio Company (Hanbio, Shanghai, China). Briefly, the AdV plasmid was digested with restriction enzymes (Fermentas, Burlington Ontario, Canada), and then the FTH1 segment was amplified and ligated to the AdV vector using DNA ligase system (Fermentas, Burlington Ontario, Canada) to generate rAdV-FTH1 plasmid. The rAdV-FTH1 plasmids were then transferred to competent E. Coli bacteria (DH5α) for transformation. The positive clones were selected and tested by gene sequence analysis.

### Transfection and collection of rAdV-FTH1 over-expressing virus

The rAdV-FTH1 over-expressing virus was generated by transfection in HEK 293 cells, and this procedure was accomplished by technical supports from Hanbio Company (Hanbio, Shanghai, China). Briefly, HEK293 cells (Hanbio, Shanghai, China) were cultured in a 60 mm dish with supplement of fresh DMEM (Hyclon) and 10% FBS (Hyclon) and incubated in a humidified atmosphere at 37°C with 5% CO_2_. When cells grew to 70–80% confluence, the culture medium was discarded and replaced with fresh DMEM containing 10% FBS, and was then ready prepared for transfection. For transfection, 2 ug rAdV-FTH1 plasmids and 4 ug backbone plasmids (pHBAd-BHG) were diluted with 300 ul DMEM at the room temperature (RT). Then 15 ul transfection reagent (LipofiterTM, Hanbio, Shanghai, China) was diluted with 300 ul DMEM at RT, and mixed with the plasmid solution. The mixed solution was stored in dark for 20 min at RT and then added to the culture dish for further incubation in a humidified atmosphere at 37°C with 5% CO_2_. After 6 hours’ incubation, the culture medium was discarded and replaced with fresh DMEM containing 10% FBS. In the following days, the cell status was examined under a light microscope every day. When cells became large, roundish and dropped from the dish, all the contents in the dish were collected and stored in a 15 ml tube. Then the tube was frozen in liquid nitrogen and thawed in 37°C water for 3 times, and then centrifuged at 3,000 rpm for 5 min at RT. The supernatant which containing the primary rAdV-FTH1 over-expressing virus (P0) was collected. For virus amplification, HEK293cells were cultured in 10 cm dishes with supplement of DMEM containing 10% FBS. When cells grew to about 80–90% confluence, 2 ml supernatant from the primary passage was added to each dish and cultured for another 2 days. When cells dropped from the dish, all the contents in the dish were collected, frozen, thawed and centrifuged as before, and the supernatant contains rAdV-FTH1 over-expressing virus of passage 1 (P1). The virus infective titer of P1 is 1×10^10^ PFU/ml as determined by modified TCID50 method.

### rAdV-FTH1 over-expressing virus infect BMSCs

BMSC collection was a gift from the key laboratory of Shantou University Medical College. All the cells used were the 3rd passage. Before infection, we designed a series of different virus concentration to get the optimal multiplicity of infection (MOI) first. The virus concentration ranged from 1.0–3.5×10^7^ PFU/ml as normalized by fresh DMEM and the corresponding MOI was marked as 100, 150, 200, 250, 300 and 350, respectively. BMSCs (1×10^5^) were seeded in a 24-well plate, when cells grew to about 30–40% confluence, 1 ml rAdV-FTH1 over-expressing virus solution of different MOI was added to each well. For the control group, 1 ml adenovirus solution of corresponding MOI was added. The plate was then placed in a humidified incubator at 37°C with 5% CO_2_. In the following 4 days, the cell status was determined under a fluorescence microscope (Olympus IX70) every day. The well that has the highest infection efficiency and the best cell status was considered as the optimal MOI. In the following experiments, BMSCs_-FTH1_ and control BMSCs were either cultured in 6-well plates, 96-well plates or 25 ml flasks. When cells grew to about 30–40% confluence, virus solution of the optimal MOI was added to the plates or flasks with supplement fresh DMEM and 10% FBS. After 72 hours, the culture medium was discarded and replaced with fresh DMEM supplied with 10% FBS. Cells were then prepared for the following determinations or other use.

### Cell viability and proliferation determination

Cell viability was determined by Trypan Blue exclusion. Briefly, BMSCs_-FTH1_ and control BMSCs were seeded in 6-well plates. When cells grew to 80–90% confluence, cells were digested by 0.25% trypsin, collected and stained with 0.4% trypan blue solution, then observed under a light microscope (Leica DMi1). Cell proliferation was assessed by MTT method according manufacturer’s instructions. In brief, BMSCs_-FTH1_ and control BMSCs were seeded in 96-well plates. When cells grew to 70–80% confluence, 10 ul MTT solution was added to each well and incubated for 4 hours in a humidified incubator at 37°C with 5% CO_2_.Then the solution was replaced with 100 ul DMSO and further incubated for 10 min. The absorbance of optical density (OD) at 490 nm was measured by a spectrophotometer.

### Induction of adipogenic and osteogenic differentiation

For adipogenic differentiation, BMSCs_-FTH1_ and control BMSCs were cultured in 6-well plates with supplement of the adipogenic differentiation medium (DMEM supplied with 10% FBS, 50 ug/ml of ascorbate-1 phosphate, 10^-7^M dexamethasone, 50 ug/ml indomethacin). For osteogenic differentiation, MSCs_-FTH1_ and control MSCs were cultured in 6-well plates with supplement of the osteogenic differentiation medium (DMEM supplied with 10% FBS 50 ug/ml ascorbate-2 phosphate, 10^−8^ M dexamethasone, 10 mM β-glycerophosphate). The medium was changed every 3 days. After 3 weeks’ incubation, the adipogenic groups were stained with Oil Red O, while the osteogenic groups were stained with alkaline phosphatase histochemistry kit according to the manufacturer’s instructions.

### Cell immunocytochemistry

For cell immunocytochemistry determination, BMSCs_-FTH1_ and control BMSCs were cultured in 6-well plates. When cells grew to about 70–80% confluence, the culture medium was discarded and washed twice with PBS. Cells were then fixed with 4% paraformaldehyde for 10 minutes at RT and washed twice with PBS, and then incubated in 3% hydrogen peroxide for 20 min at RT. After that, 1% blocking solution agent (BSA) was added to each well and incubated for another 30 min at RT. Cells were then incubated with the primary antibody (rabbit anti-rat feritin heavy chain polyclonal antibody) in 1% BSA at 37°C for 2 h and washed twice with PBS, and the second antibody (goat anti-rabbit, HRP) in 1% BSA was added and incubated for another 1 h at RT. The cells were then blocked with goat serum for 30 min and stained with DAB and hematoxylin for 2 min, then washed with 1% hydrochloric acid and deionized water for 3 times, and observed under a light microscope (Leica DMi1).

### ELISA assay

For ELISA assay determination, BMSCs_-FTH1_ and control BMSCs were cultured in 25 ml flasks. When cells grew to 70–80% confluence, the culture medium was discarded and washed twice with ice-cold PBS (pH 7.2–7.4). The cells were then trypsinized, normalized to 10×10^6^/ml and collected in 2 ml EP tubes. The tubes were frozen in -20°C overnight and thawed in 37°C water twice to break down the cell membrane, and then centrifuged at 5000 rpm, 4°C for 5 min. The supernatant was collected and prepared for ELISA determination using Mouse ferritin heavy chain ELISA kit (CUSABIO, Wuhan, China) according to the manufacturer’s instructions. Every group has 3 samples and every sample was test 3 times to get an average.

### MRI scans of cell suspension

MRI scan was performed at the time point of1, 2, 3 and 4 weeks after virus infection. Firstly, BMSCs_-FTH1_ and control BMSCs were collected and suspended in 2 ml agarose (37°C, 1% PBS) in EP tubes, and the cell number was normalized to 1×10^6^ in each tube. The EP tubes were then fixed in a 50 ml beaker filled with cooled agarose (1% PBS). MRI scan was performed on a 7 T MRI device (Angilent, 160AS) equipped with a 2-channel body coil (Varian, 9563). Each test and control group had 5 samples, and every sample was scanned 3 times. The scanning parameters were as follows, fast spin-echo pulse sequence with 8 echoes (TE = 4, 21, 30, 44, 65, 94, 137,199 ms), TR = 2000 ms, thickness = 2 mm, and the total time was 19 min 50 s. The T2 mapping images were reconstructed automatically by pixel-based fit software soon after scanning finished. A 5×5 mm region of interest (ROI) was placed in the center of T2 mapping images to calculate the T2 values. Every tube was calculated 3 times before an average was made. In calculating the T2 relaxation time (R2) andδR2, the latter of which was defined as the ratio of change in R2 between test and control groups, the following formula was used to respectively. *R2 = 1/T2*,*δR2 = (R*_*BMSCs-FTH1*_*-R*_*MSCs*_*)/R*_*BMSCs*_*×100%*.

### Statistical analysis

Statistical analysis was performed using SPSS version 17.0 software (SPSS Inc., Chicago, IL, USA). Data are summarized as means±standard deviation (SD). The comparison between multiple groups was analyzed by one-way analysis of variance (ANOVA), and that between each two groups was processed by independent-sample t test, with p<0.05 being considered as statistically significant. The correlations between OD value, ferritin concentration, R2and δR2 were analyzed by Pearson correlation.

## Results

### 1. Verification of rAdV-FTH1 over-expressing virus

**Fig 1:**
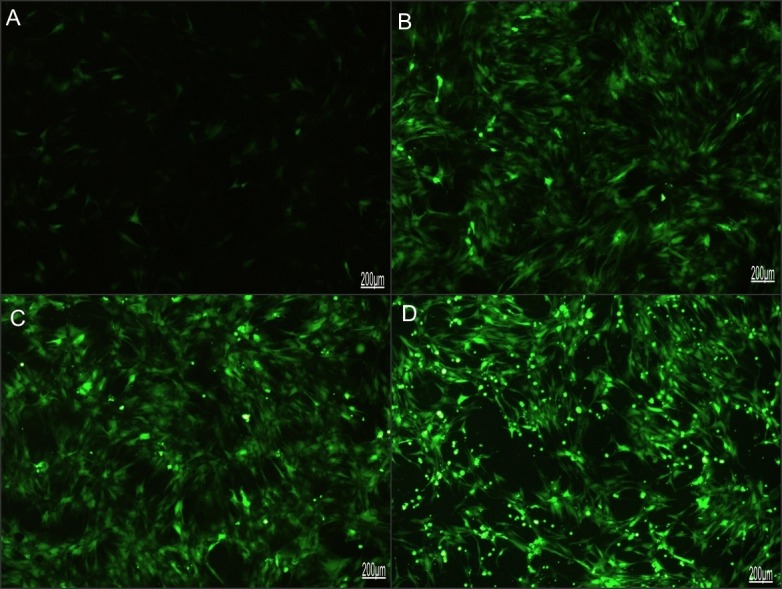
GFP gene expression in BMSCs confirmed by fluorescence microscope. (A-D) 24h, 48h, 72h and 96h after rAdV-FTH1 over-expressing virus (MOI: 150) infect BMSCs. GFP expression reached the peak in 72 hours as detected by fluorescence microscope.

The construction of rAdV-FTH1 over-expressing virus was verified by PCR and gene sequence analysis. The results of PCR showed the monoclone of rAdV-FTH1 product had about 750bp of nucleotides (S1 Fig), and the reconstructed rAdV-FTH1 plasmid, as revealed by the gene sequence analysis, had the matching sequence with the open reading frame (ORF) of FTH1 gene (S2 and S3 Figs), indicating the success of construction. After transfection and amplification in HEK 293 cells, the final virus infective titer of rAdV-FTH1 over-expressing virus was 1.0×10^10^ PFU/ml as determined by modified TCID50 method. The optimal MOI for infection was 150 and the corresponding virus titer was 1.5×10^7^ PFU/ml. As confirmed by fluorescence microscope, the GFP gene was highly expressed in BMSCs and reached the peak in 72 hours after virus infection, indicating that rAdV-FTH1 over-expressing virus has entered the target cells (Fig 1).

### 2. Effect of ferritin over-expression on cell viability, proliferation and multi-differentiation

**Fig 2:**
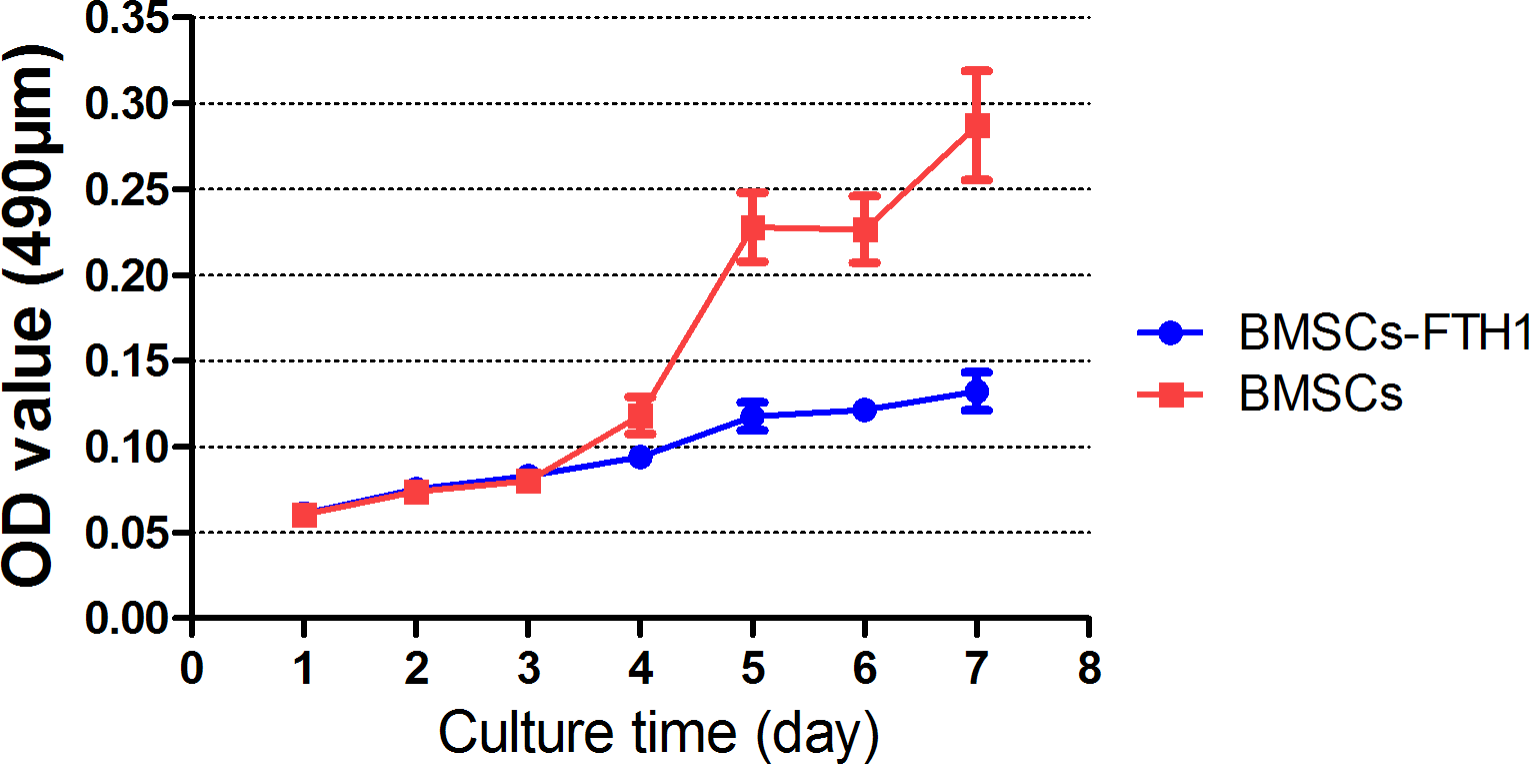
Growth curve of BMSCs_-FTH1_ and control BMSCs. Cell growth curve showed no difference between BMCs_-FTH1_ and control BMSCs in the first 3 days but BMCs_-FTH1_slowed down in the last 4 days.

**Fig 3:**
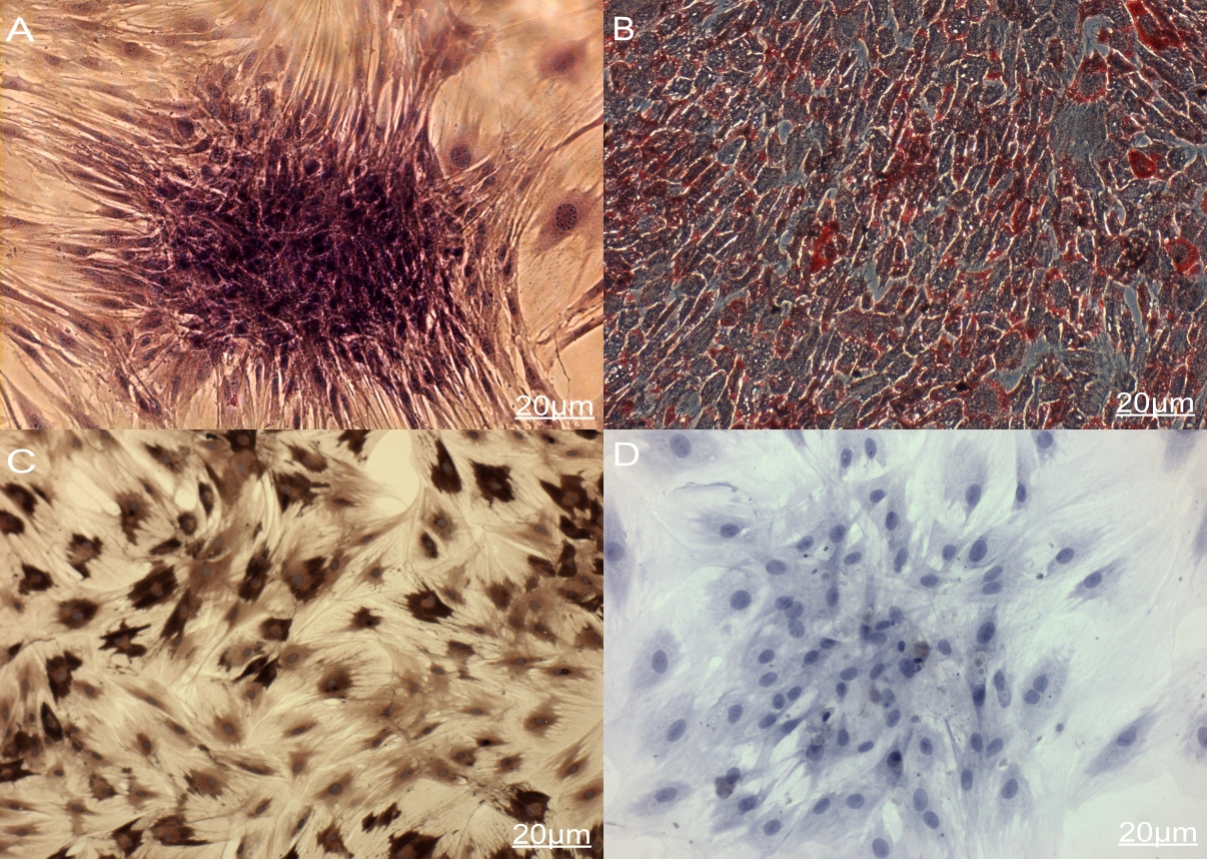
Multilineage differentiation and ELLSA assay. (A) BMSCs_-FTH1_ induced osteogenic differentiation formed alkaline phosphatase-positive aggregated nodules. (B) BMSCs_-FTH1_ induced adipogenic differentiation showed positive Oil red O stained. (C) BMSCs_-FTH1_ were positive to anti-rat ferritin heavy chain polyclonal antibody and stained yellow to dark brown, (D) Control BMSCs were negative stained to blue color.

In terms of the cell viability, no difference was found between BMSCs_-FTH1_ and control BMSCs as determined by Trypan Blue exclusion. And in terms of the cell proliferation, as determined by MTT and the growth curve, no difference was found in the first 3 days but the proliferation rate of BMSCs_-FTH1_ slowed down in the last 4 days compared to that among the control BMSCs (Fig 2). As far as multilineage differentiation is concerned, after 3 weeks’ culture in osteogenic differentiation medium, both BMSCs_-FTH1_ and control BMSCs formed alkaline phosphatase-positive aggregated nodules, indicating that MSCs differentiated into osteoblast cells (Fig 3A). And in terms of adipogenic differentiation, both BMSCs_-FTH1_ and control BMSCs were positively stained by Oil red O, indicating that the cells could differentiate into adipose cells (Fig 3B).

### 3. Cell immunocytochemistry and ELISA assay

**Fig 4:**
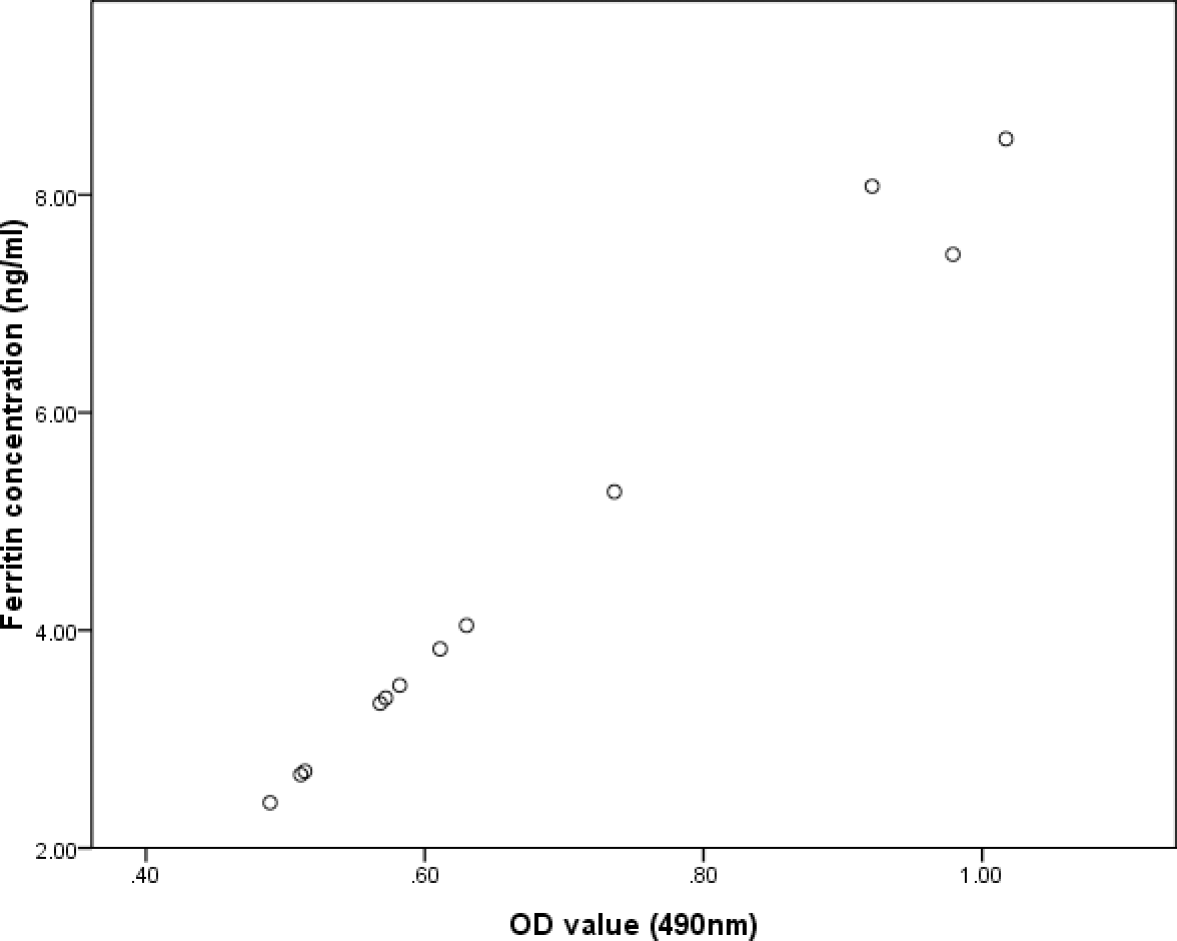
Correlation of OD value and ferritin concentration. The change in the OD value and ferritin concentration was positively correlated, with statistical significance (r = 0.992, p<0.01).

As confirmed by the cell immunocytochemistry, BMSCs_-FTH1_were positive to anti-rat ferritin heavy chain polyclonal antibody. Positive cells were stained brown yellow to dark brown color (Fig 3C), while the control BMSCs were stained blue by hematoxylin (Fig 3D). In order to determine the ferritin concentration by ELISA assay, a standard linear curve and corresponding formula (y = 0.0866x+0.2798) were generated first by testing OD values of the standard sample. Then the OD values of the test samples were taken into the formula to figure out corresponding ferritin concentrations (Table 1). As seen in the table, OD values gradually decreased during 4 weeks’ observation, with the difference between the groups having statistical significance (F = 71.9, p<0.001). Ferritin concentration also gradually decreased, with the difference between groups being statistically significant (F = 74.34, p<0.01). The relation between the OD value and ferritin concentration is significant positively correlated (r = 0.992, p<0.01) as determined by Pearson Correlation (Fig 4).

**Table 1:** The OD value, ferritin concentration (ng/ml), R2 value andδR2 at different time points.

	1w	2w	3w	4w
OD	0.927±0.048	0.659±0.067	0.574±0.072	0.504±0.190
Con.	8.014±0.533	4.381±0.778	3.399±0.085	2.598±0.158
R2_BMSCs- FTH1_	16.65±1.28	15.63±1.37	15.53±0.88	14.61±1.28
R2_BMSCs_	13.99±0.80	13.87±0.83	14.25±0.53	13.69±1.03
δR2	15.84±2.47	11.36±4.06	8.15±2.96	6.20±2.81

### 4. MRI results

**Fig 5:**
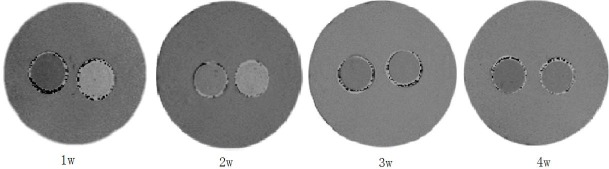
MRI results of BMSCs_-FTH1_ and BMSCs on 7T MRI device. (A) As revealed by the T2-mapping images of BMSCs_-FTH1_ and control BMSCs acquired by 7T MRI, the signal intensity of BMSCs_-FTH1_ (left) was darker than that of BMSCs (right).

**Fig 6:**
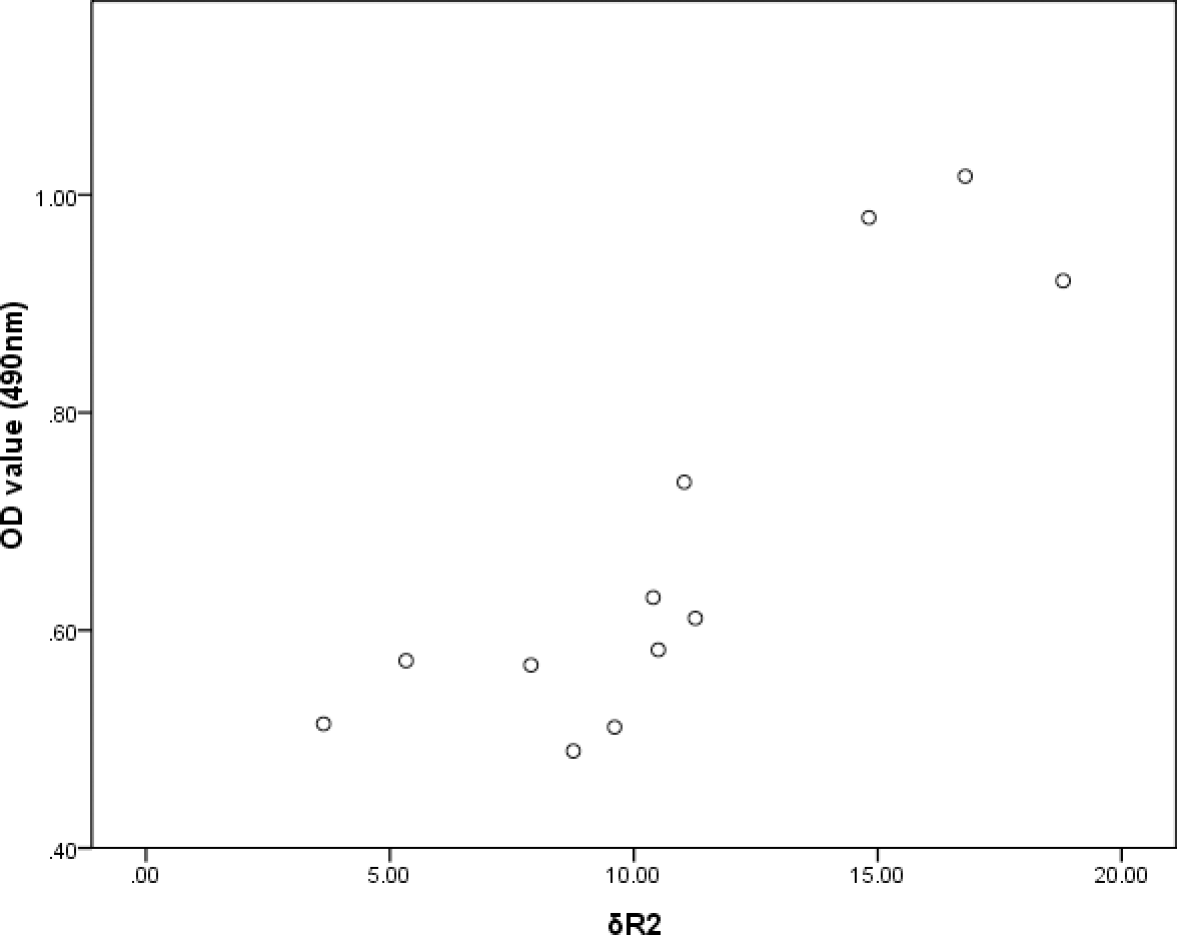
Correlation of δR2 and OD value. The change in δR2 and OD value was positively correlated, with statistical significance (r = 0.876, p<0.01).

**Fig 7:**
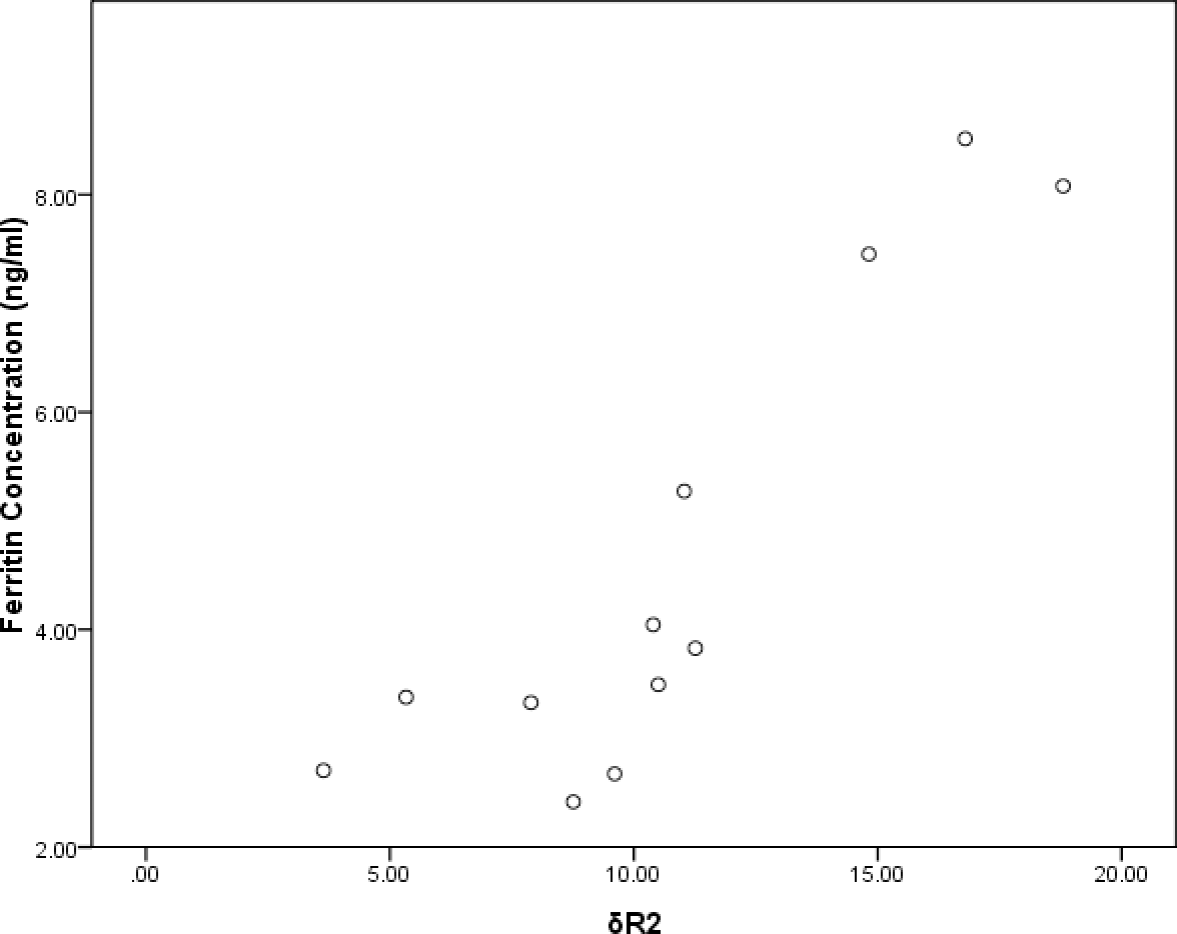
Correlation ofδR2 and ferritin concentration. The change in δR2 and ferritin concentration was positively correlated, with statistical significance (r = 0.899, p<0.01).

**Fig 8:**
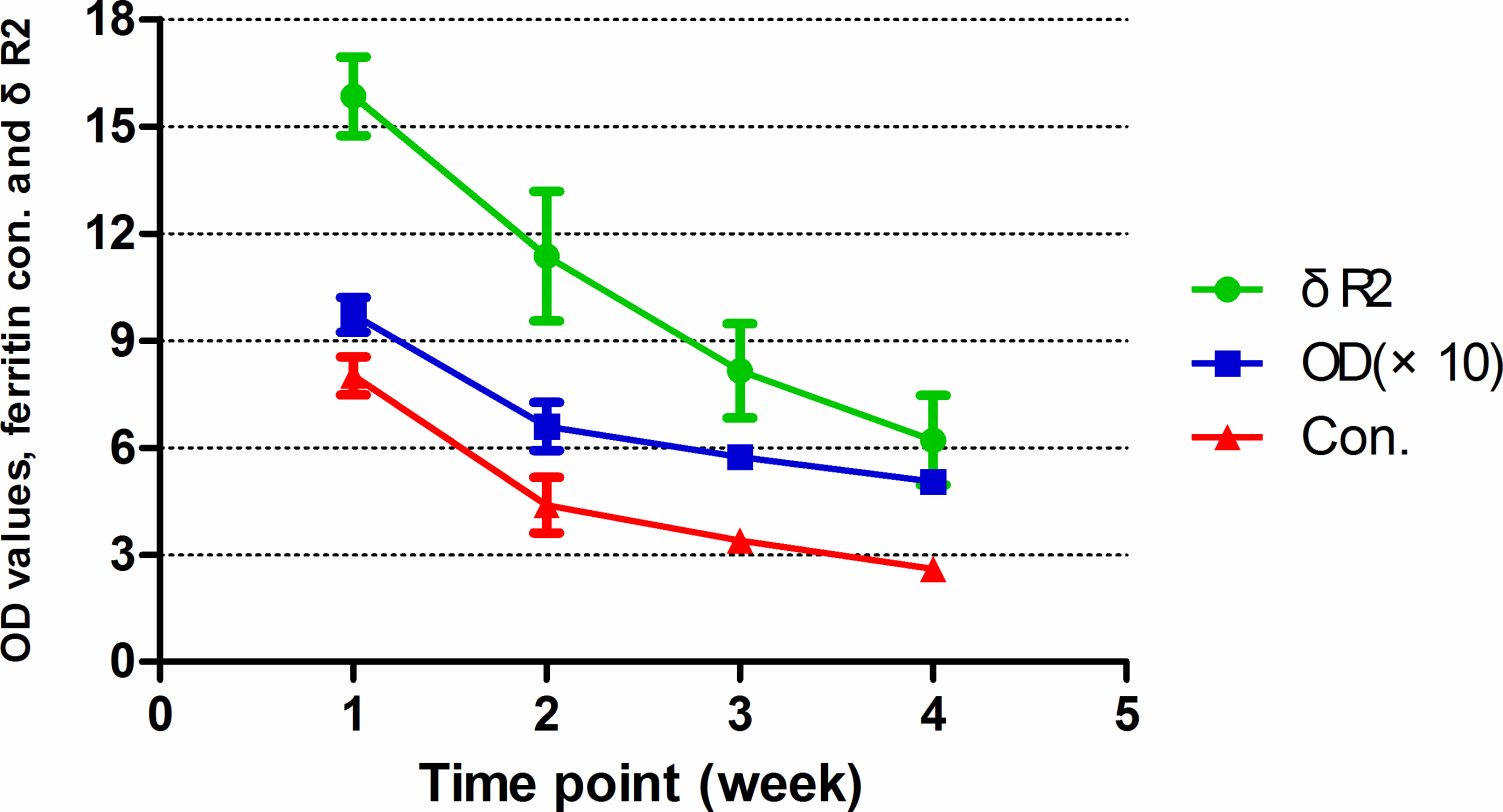
The trend of OD, ferritin concentration and δR2. OD value, ferritin concentration andδR2 gradually decreased during 4 weeks’ observation.

T2 values at different time points were calculated from the T2 mapping images of each tube, then R2 values andδR2 were calculated with corresponding formulas. The R2 values of BMSCs_-FTH1_ vs control BMSCs at 1–4 weeks’ time points were 16.65±1.28 s^-1^ vs 13.99±0.80 s^-1^, (t = 3.94, p = 0.004), 15.63±1.37 s^-1^ vs 13.87±0.83 s^-1^ (t = 2.47, p = 0.039), 15.53±0.88 s^-1^ vs 14.25±0.53 s^-1^ (t = 2.80, p = 0.023) and 14.61±1.28 s^-1^ vs 13.69±1.03 s^-1^ (t = 1.25, p = 0.24), respectively. As shown in the T2 mapping images acquired by the 7T MR device, the tubes containing BMSCs_-FTH1_ showed darker gray scale than that containing the control BMSCs (Fig 5). During 4 weeks’ time,δR2 gradually decreased, with the difference between the groups having statistical difference (F = 9.04, p<0.001).δR2 was highly correlated with the OD values (r = 0.876, p<0.01) and the ferritin concentration (r = 0.899, p<0.01), as determined by Pearson correlation (Figs 6 and 7). The trend of OD values, ferritin concentration and δR2 are illustrated in Fig 8.

## Discussion

Cell labeling and monitoring are important parts in stem cell applications. Among so many innovative labeling strategies, the endogenous ferritin over-expression method is an attractive one. In the present study, we confirmed that ferritin could be over-expressed in BMSCs through exogenous rAdV-mediated FTH1 gene over-expression. Ferritin accumulated in BMSCs could be detected by 7T MRI device in vitro. Ferritin over-expression in BMSCs results a decrease in T2 signals and darker gray scales on T2-mapping images compared to that of the control BMSCs. And ferritin concentration was positively correlated withδR2 in MRI. These results indicated that the endogenous ferritin generated by rAdV mediated FTH1 gene over-expression could serve internal MRI contrast for BMSCs.

Our work showed that ferritin over-expression did not interfere with cell viability and pluripotent differentiation of BMSCs, which is consistent with previous studies [19–21]. The cell proliferation rate, as shown in MTT results, slowed down due to ferritin over-expression. This result is line with the study of Cozzi A, who found that ferritin ferroxidase might hinder the proliferation of Hela cells [22], but inconsistent with some other studies partially which assert that ferritin does not interfere with cell viability, proliferation and multi-differentiation [15, 19, 23]. It was reported that ferritin over-expression can increase net intracellular iron content, and intracellular iron increase can produce reactive oxygen species through Harber–Weiss/Fenton reactions, which result in lipid, protein and DNA damage and decrease of cell viability and proliferation[24–26]. The gap between different studies suggests that there are variability among different cell types in sensitivity to ferritin over-expression and change in iron homeostasis.

As seen in MRI results, a T2 signal decrease was found on T2 mapping images as a result of ferritin over-expression. The tubes containing BMSCs_-FTH1_ had darker gray scale than those of containing control BMSCs. It is known that ferritin over-expression can transiently decrease iron concentration in cells, induce iron atom redistribution as well as compensatory uptake of iron atoms from the surrounding environments, and result in net increase in iron content eventually [27, 28]. Compared with control BMSCs,δR2 of BMSCs_-FTH1_ increased as much as 16% in the first week and 6% till the fourth week. Although it is hard to distinguish the difference in the gray scale visually in the last two weeks, difference was observed statistically between R2 values as determined by T2 mapping sequence, suggesting that MRI can detect very small signal change even at a low ferritin concentration. As ferritin content gradually decreased during 4 weeks’ observation, we found a good correlation between OD and R2 value throughout the study. The results by MRI and ELISA reveals that δR2 also has good linear correlation with ferritin contents. These exciting results indicate that it may be feasible to estimate cell deposition and status via the internal contrast generated by ferritin over-expression quantitatively [17, 29]. Ferritin may open many possibilities in tracking cell recruitments and migrations, as well as in disease models and gene activations [27, 28, 30]. Furthermore, unlike any other exogenous administrated contrasts, ferritin exists only in living cells and less vulnerable to interference from macrophages or other organelles, thus the change in ferritin content is a direct symbolization of cell status, which may provide more accurate information for cell monitoring.

The adenovirus vector, which can infect both dividing cells and resting cells, has been wildly used in both laboratory and clinical trials. Compared to some other deliver vehicles like lentiviral vector or retrovirus vector, adenovirus vector has extraordinary advantages in its safety and high efficiency [31–33]. However, the exogenous genes mediated by adenovirus vector cannot be integrated into the host chromosome of BMSCs and can only be expressed in cell cytoplasm [34]. As cells divide, any intracellular labels, including iron-oxide particles or ferritin-stored irons, would be diluted. This special feature of adenovirus vector should be taken into consideration before study design. And in this regard, the adenovirus vector may not be suitable for long-term gene expression. That is why we found a gradual decrease of ferritin content and corresponding MRI signals in our study. Since the original purpose of our work was to make clear whether rAdV mediated FTH1 gene can be over-expressed in BMSCs and to figure out the relationship between ferritin content and MRI signals in a dynamic course rather than long-term monitoring, it is reasonable to choose a short-term vector in this regard. The rAdV vehicle may open many possibilities in short-term administration of exogenous genes or probes for labeling or treatment, since the insertion site for FTH1 gene may also be linked with other genes. It is believed that the choice of delivery vehicles depends on the design and purpose of the study. We do admit a continuous and stable production of ferritin in daughter cells is essential for long-term gene expression and cell labeling. If studies were designed for long-term administration, other delivery vehicles like the lentiviral vector, which was reported can maintain target gene expression for up to 6 month, are highly recommended [20].

Our study has several limitations. Firstly, since our study was not designed for long-term monitoring, the long-term effect of rAdV mediated ferritin over-expression on BMSCs is unknown. Secondly, only in vitro study was performed so far, whether rAdV mediated ferritin could be monitored in vivo need to be further explored. Besides, MRI is still not sensitive enough to detect a very small amount of ferritin content, as a certain number of cells are needed to reach the detectable threshold even in the ultra-high field MRI device. MRI signal is the consequence of a collective phenomenon rather than a single cell. When applied to in vivo studies, MRI is challenged by the difficulties in distinguishing ferritin from other confusing cells, tissues or organs. In this respect, we should endeavor to design better transgenic vectors, and improve MRI sensitivity and accuracy for bright prospects of endogenous ferritin reporter in stem cell applications.

## Supporting information

S1 Fig Verification of rAdV-FTH1 construction by DNA ladder and PCR. From left to right: Line 1: DNA marker of GeneRay 1kb DNA Marker (from up down: 12000bp, 8000bp, 6000bp, 5000bp, 4000bp, 3000bp, 2500bp, 2000bp, 1500bp, 1000bp, 750bp, 500bp, 250bp). Line 2: line of pHBAd-MCMV-GFP vector. Line 3: DNA marker (from up down: 4500, 3000, 2000, 1200, 800, 500, 300, 100bp). Line 4: PUC57-FTH1 EcoRI/BamHI cutted sequence. Line 5: GeneRay 250bp DNA Ladder (from up down: 1000bp, 750bp, 500bp, 250bp, 100bp). Line 6: monoclonal PCR product of FTH1.
(TIF)

S2 Fig Gene sequencing result of the constructed FTH1 sequence (in PUC57). (TIF)

S3 Fig Gene sequence analysis of the recontructed rAdV-FTH1virus plasmid. Result shows the comparison of gene sequence of rAdV-FTH1 (upper line) and the target gene ORF sequence (lower line). Sequences in blue color are the matching parts.
(TIF)

S4 Fig The rAdV-FTH1 over-expressing virus were harvested by infecting HEK293 cells. (TIF)

S1 Table The OD value of MSCs-FTH1 and control BMSCs by MTT. (DOCX)

S2 Table OD values of the standard sample from the Mouse ferritin heavy chain ELISA kit. (DOCX)

S3 Table OD value of the test samples and corresponding ferritin concentration at different time points. (DOCX)

S4 Table The relaxation rate (R2) of BMSC-FTH1 and control BMSCs at different time points. (DOCX)
