# Supplementary figures and images for: Adenoviral vector mediated ferritin over-expression in mesenchymal stem cells detected by 7T MRI in vitro
Source: PLoS One. 2017 Sep 25;12(9):e0185260. doi: 10.1371/journal.pone.0185260 (PMC5612726; doi:10.1371/journal.pone.0185260)

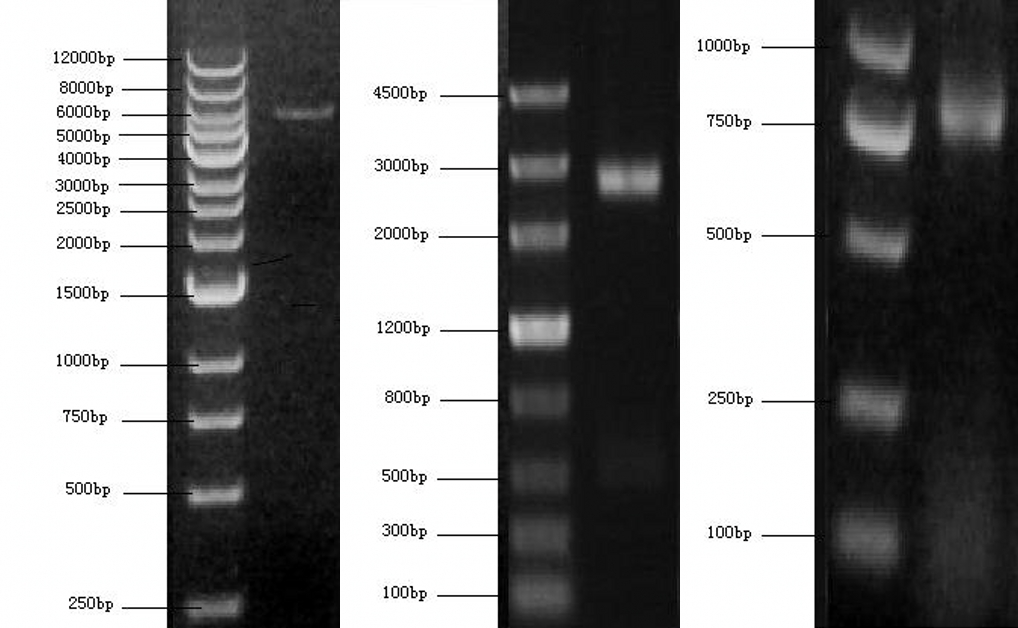

Supplement: S1 Fig — From left to right: Line 1: DNA marker of GeneRay 1kb DNA Marker (from up down: 12000bp, 8000bp, 6000bp, 5000bp, 4000bp, 3000bp, 2500bp, 2000bp, 1500bp, 1000bp, 750bp, 500bp, 250bp). Line 2: line of pHBAd-MCMV-GFP vector. Line 3: DNA marker (from up down: 4500, 3000, 2000, 1200, 800, 500, 300, 100bp). Line 4: PUC57-FTH1 EcoRI/BamHI cutted sequence. Line 5: GeneRay 250bp DNA Ladder (from up down: 1000bp, 750bp, 500bp, 250bp, 100bp). Line 6: monoclonal PCR product of FTH1. (TIF) [file pone.0185260.s001.tif]

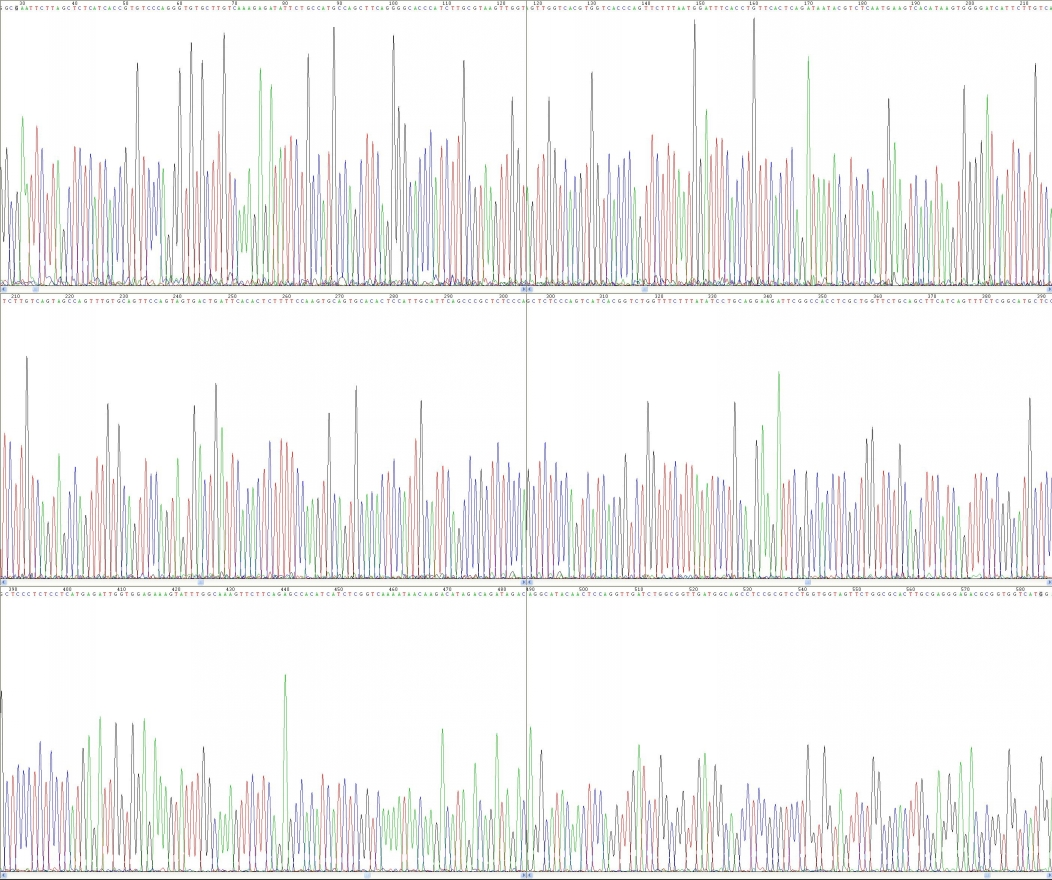

Supplement: S2 Fig — (TIF) [file pone.0185260.s002.tif]

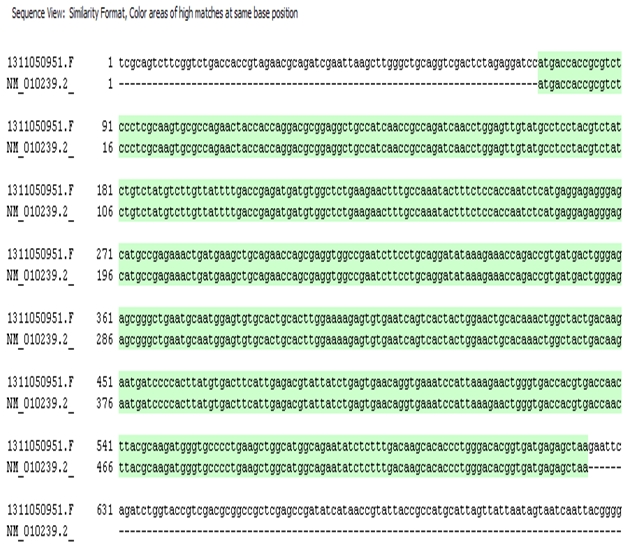

Supplement: S3 Fig — Result shows the comparison of gene sequence of rAdV-FTH1 (upper line) and the target gene ORF sequence (lower line). Sequences in blue color are the matching parts. (TIF) [file pone.0185260.s003.tif]

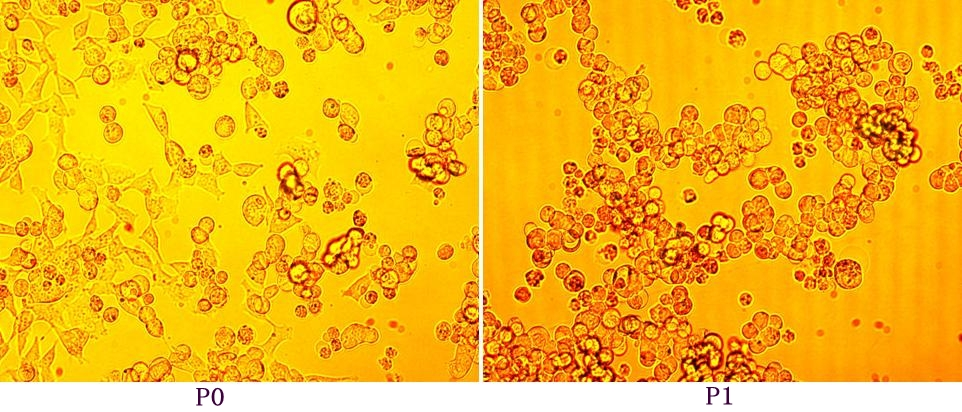

Supplement: S4 Fig — (TIF) [file pone.0185260.s004.tif]
