# Supplementary material for: Adenoviral vector mediated ferritin over-expression in mesenchymal stem cells detected by 7T MRI in vitro
Source: PLoS One. 2017 Sep 25;12(9):e0185260. doi: 10.1371/journal.pone.0185260 (PMC5612726; doi:10.1371/journal.pone.0185260)
Supplement: S1 Table — (DOCX) [file pone.0185260.s005.docx]

**S1 Table. The OD value of MSCs-FTH1 and control BMSCs by MTT**

|  | BMSC_-FTH1_ | | | | BMSC | | | |
| --- | --- | --- | --- | --- | --- | --- | --- | --- |
|  | A | B | C | M±SD | A | B | C | M±SD |
| 1d | 0.0634 | 0.0634 | 0.0562 | 0.061±0.0041 | 0.0608 | 0.0624 | 0.0586 | 0.0606±0.0041 |
| 2d | 0.0778 | 0.0714 | 0.0766 | 0.0757±0.0034 | 0.076 | 0.0712 | 0.075 | 0.074±0.0025 |
| 3d | 0.082 | 0.0848 | 0.0824 | 0.083±0.0015 | 0.0844 | 0.075 | 0.0804 | 0.0799±0.0047 |
| 4d | 0.0932 | 0.0942 | 0.0952 | 0.0942±0.0010 | 0.1304 | 0.1098 | 0.1144 | 0.1182±0.0108 |
| 5d | 0.1096 | 0.1258 | 0.1178 | 0.1177±0.0081 | 0.2064 | 0.246 | 0.2316 | 0.228±0.020 |
| 6d | 0.1214 | 0.1212 | 0.1216 | 0.1214±0.0002 | 0.2272 | 0.2068 | 0.2456 | 0.2265±0.0194 |
| 7d | 0.1446 | 0.1292 | 0.1232 | 0.1323±0.01103 | 0.2852 | 0.2564 | 0.320 | 0.2872±0.3183 |

OD values were tested at 490 nm by a spectrophotometer.
