# Supplementary material for: Adenoviral vector mediated ferritin over-expression in mesenchymal stem cells detected by 7T MRI in vitro
Source: PLoS One. 2017 Sep 25;12(9):e0185260. doi: 10.1371/journal.pone.0185260 (PMC5612726; doi:10.1371/journal.pone.0185260)
Supplement: S2 Table — (DOCX) [file pone.0185260.s006.docx]

**S2 Table. OD values of the standard sample from the Mouse ferritin heavy chain ELISA kit**

| Concentration of the Standard sample（ng/ml） | | | | | | | | |
| --- | --- | --- | --- | --- | --- | --- | --- | --- |
|  | 20 | 10 | 5 | 2.5 | 1.25 | 0.625 | 0.312 | 0 |
| OD-1  OD-2  OD-3  OD-Average | 1.829  1.801  1.78  1.803 | 1.407  1.433  1.425  1.422 | 1.057  0.987  0.978  1.007 | 0.551  0.56  0.564  0.558 | 0.308  0.303  0.3  0.304 | 0.222  0.218  0.219  0.219 | 0.19  0.188  0.187  0.188 | 0.172  0.173  0.175  0.173 |
