# Supplementary material for: Adenoviral vector mediated ferritin over-expression in mesenchymal stem cells detected by 7T MRI in vitro
Source: PLoS One. 2017 Sep 25;12(9):e0185260. doi: 10.1371/journal.pone.0185260 (PMC5612726; doi:10.1371/journal.pone.0185260)
Supplement: S3 Table — (DOCX) [file pone.0185260.s007.docx]

**S3 Table. OD value of the test samples and corresponding ferritin concentration at different time points**

|  | Sample 1  OD Con. | | Sample 2  OD Con. | | Sample 3  OD Con. | |
| --- | --- | --- | --- | --- | --- | --- |
| 1w  2w  3w  4w | 1.017  0.736  0.582  0.511 | 8.5127  5.2717  3.4935  2.6736 | 0.979  0.63  0.572  0.514 | 8.0777  4.0439  3.3779  2.7044 | 0.921  0.611  0.568  0.489 | 7.4519  3.8283  3.3279  2.4157 |

OD values were tested at 490 nm by a spectrophotometer.

Con. (ng/ml)
