# Supplementary material for: Adenoviral vector mediated ferritin over-expression in mesenchymal stem cells detected by 7T MRI in vitro
Source: PLoS One. 2017 Sep 25;12(9):e0185260. doi: 10.1371/journal.pone.0185260 (PMC5612726; doi:10.1371/journal.pone.0185260)
Supplement: S4 Table — (DOCX) [file pone.0185260.s008.docx]

**S4 Table. The Relaxation rate (R2) of BMSC-FTH1 and control BMSCs at different time points**

|  |  | 1 | 2 | 3 | 4 | 5 | M±SD |
| --- | --- | --- | --- | --- | --- | --- | --- |
| 1w  2w  3w  4w | R2_BMSCs-FTH1_  R2_BMSCs_  δR2  R2_BMSCs-FTH1_  R2_BMSCs_  δR2  R2_BMSCs-FTH1_  R2_BMSCs_  δR2  R2_BMSCs-FTH1_  R2_BMSCs_  δR2 | 18.40  15.31  16.79  16.95  15.08  11.03  16.76  15.00  10.50  16.76  15.15  9.61 | 15.79  13.45  14.82  15.87  14.22  10.40  15.38  14.56  5.33  14.02  13.51  3.64 | 17.34  14.08  18.80  14.83  13.16  11.26  15.08  13.89  6.89  13.70  12.5  8.76 | 16.57  13.82  16.60  16.81  13.83  17.73  15.96  14.08  11.78  14.78  14.22  3.69 | 15.15  13.30  12.21  13.70  12.82  6.42  14.46  13.70  5.26  13.77  13.05  5.23 | 16.65±1.28  13.99±0.80  15.84±2.47 15.63±1.37 13.87±0.83  11.36±4.06 15.53±0.88  14.25±0.53  8.15±2.96 14.61±1.28  13.69±1.03  6.20±2.81 |
